# Clinical features and risk factors of panitumumab-induced interstitial lung disease: a postmarketing all-case surveillance study

**DOI:** 10.1007/s10147-015-0834-3

**Published:** 2015-05-13

**Authors:** Masahiro Osawa, Shoji Kudoh, Fumikazu Sakai, Masahiro Endo, Tetsuya Hamaguchi, Yumiko Ogino, Miyo Yoneoka, Motonobu Sakaguchi, Hiroyuki Nishimoto, Akihiko Gemma

**Affiliations:** Pharmacovigilance Department, Takeda Pharmaceutical Company Limited, 4-9, Hiranomachi 2-chome, Chuo-ku, Osaka, 541-0046 Japan; Fukujuji Hospital, Kiyose, Japan; Japan Anti-Tuberculosis Association, Tokyo, Japan; Department of Diagnostic Radiology, Saitama International Medical Center, Saitama Medical University, Hidaka, Japan; Division of Diagnostic Radiology, Shizuoka Cancer Center, Nagaizumi-Cho, Shizuoka, Japan; Department of Gastrointestinal Medical Oncology, National Cancer Center Hospital, Tokyo, Japan; Department of Pulmonary Medicine and Oncology, Graduate School of Medicine, Nippon Medical School, Tokyo, Japan

**Keywords:** Colorectal cancer, Interstitial lung disease, Panitumumab, Postmarketing surveillance, Risk factors

## Abstract

**Background:**

Drug-induced interstitial lung disease (ILD) is one of the most serious adverse reactions associated with the molecularly targeted drugs. Panitumumab has been approved for advanced or recurrent colorectal cancer. Although there were no adverse reaction reports of ILD in panitumumab monotherapy, 4 cases in combination chemotherapy were reported prior to its approval in Japan in 2010. Several studies also reported that the incidence of drug-induced ILD was higher in Japan than in other countries. The clinical features of ILD and the associated risk factors therefore need investigation.

**Methods:**

We analyzed the data from 3085 unresectable, advanced or recurrent colorectal cancer patients enrolled in a postmarketing all-case surveillance study of panitumumab in Japan. ILD case reports were assessed based on the clinical and radiologic findings by a committee of external experts. Multivariate analysis using Cox’s hazard model identified the risk factors.

**Results:**

ILD incidence (1.3 %) and mortality rates (51.3 %) were similar to those of patients receiving another anti-epidermal growth factor receptor (EGFR) monoclonal antibody in Japan. No specific onset timing was determined. Although panitumumab-specific ILD findings were not observed in computed tomography images or clinical practice, panitumumab can induce ILD with diffuse alveolar damage, as do the other anti-EGFR targeting drugs. A history/complication of ILD, male sex, poor general condition, and 65 years or older were identified as ILD risk factors, and no history of previous drug treatment was an apparent risk factor.

**Conclusion:**

Panitumumab-induced ILD can occur at any time after initiation, and close and regular monitoring is needed.

## Introduction

Panitumumab is a high-affinity, fully human monoclonal antibody targeting epidermal growth factor receptor (EGFR) [1]. Panitumumab was first approved for the treatment of patients with EGFR-expressing metastatic colorectal cancer as monotherapy in the USA in 2006, based on the results of a multinational, open-label, randomized phase III study showing an improvement in the median progression-free survival [2].

In Japan, panitumumab was approved in 2010 for the treatment of wild-type *KRAS* unresectable, advanced or recurrent colorectal cancer as monotherapy, and for use in combination therapy in all-line treatment settings based on the global clinical trials and a Japanese phase II trial [3–9]. As a condition for its approval, the Japanese Ministry of Health, Labour and Welfare requested the implementation of a postmarketing all-case surveillance study to confirm the safety and efficacy of panitumumab in the clinical setting because the number of Japanese patients enrolled in the global and Japanese clinical trials was limited. Hence, the postmarketing all-case surveillance study was conducted in Japan. Following is a summary of the survey results from 3085 enrolled patients [10]: (a) the favorable toxicity profile and clinical benefit of panitumumab treatment in daily clinical practice were confirmed, and were similar to those reported in the previous clinical trials; and (b) the most common adverse drug reaction observed was skin disorders (78.4 %), including dermatitis acneiform, paronychia, dry skin, and pruritus, followed by electrolyte abnormalities (19.3 %), infusion reaction (1.5 %), interstitial lung disease (ILD) (1.3 %), and cardiac disorders (0.2 %).

Drug-induced ILD is noted as one of the most serious adverse reactions associated with molecular targeting agents including an anti-EGFR monoclonal antibody (cetuximab) and EGFR-tyrosine kinase inhibitors (TKIs) (gefitinib and erlotinib), as it can be fatal [11–20]. Multiple studies have reported that the incidence of drug-induced ILD was higher in Japan than in other countries, and this trend was more prominently observed in the postmarketing surveillance studies of the EGFR-TKIs [13–15, 21] and anti-EGFR antibody [20].

There were no adverse drug reaction reports of ILD in panitumumab monotherapy (1052 patients) prior to its approval in Japan; however, such reports were received in combined therapy with FOLFOX4 (fluorouracil, leucovorin, and oxaliplatin) [0.6 % (2/322 patients)] and with FOLFIRI (fluorouracil, leucovorin, and irinotecan) [0.7 % (2/302 patients)] [10]. In addition, during the course of clinical trials, a patient with non-small-cell lung cancer who had a history of pulmonary fibrosis developed ILD and died. Thereafter, patients with a history of or current ILD were excluded from clinical trials, and the experience of administration to patients with a history of ILD was limited. It is therefore important to evaluate the clinical features and risk factors of panitumumab-induced ILD to prevent the fatal outcome of ILD as well as to ensure an appropriate use of the drug.

## Materials and methods

### Patients and surveillance design

This postmarketing surveillance study was planned to include all patients treated with panitumumab (Vectibix) from the start date (June 15, 2010) of its launch in Japan (ClinicalTrials.gov: NCT02089737; Japan Pharmaceutical Information Center–Clinical Trials Information: 132374) [10].

To promote appropriate use and evaluate safety information, a Vectibix Appropriate Use Committee, a Vectibix Safety Evaluation Committee, and a Vectibix ILD review subcommittee were organized. The Vectibix ILD review subcommittee was established to evaluate the relationship between panitumumab and ILD, or the tendency of ILD occurrence, from the viewpoint of a third party on the basis of information on the treatment of patients in whom ILD developed or who had symptoms or disease states related to ILD after they received panitumumab. The registration period of this postmarketing surveillance study was from June 2010 to November 2010.

All patients were registered by fax before their first administration of panitumumab after panitumumab was marketed. Regarding ILD risk, a letter recommending to avoid administration was sent from the Vectibix Appropriate Use Committee if the patient had a history of ILD or pulmonary fibrosis and previous or concurrent ILD with the FOLFIRI regimen. A letter recommending to reconsider the administration was sent if the patient had a history of ILD.

Data on patients’ demographics, clinical course, and safety information were collected using a case report form at 10 months after the start of the panitumumab therapy or at the time when the therapy was discontinued for any reason within less than 10 months. Reported terms were classified according to the Preferred Terms in the Medical Dictionary for Regulatory Activities (MedDRA), version 15.0.

### Evaluation of ILD

**Fig. 1:**
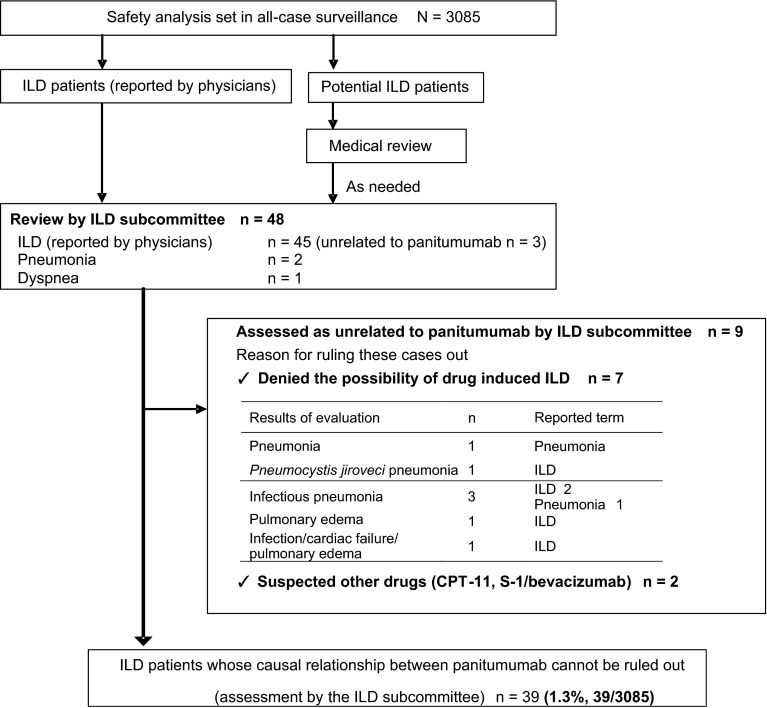
Diagnosis of ILD. ILD cases were evaluated and determined according to the flowchart. The ILD review subcommittee assessed each ILD case report based on the clinical and radiographic findings. *ILD* interstitial lung disease

The Vectibix ILD review subcommittee (hereafter referred to as the committee) was established before this survey was performed. The committee consisted of the following 5 external experts to assess ILD case reports appropriately: 2 experts in radiology, 2 in pulmonology, and 1 in medical oncology. ILD and potential ILD case reports were then evaluated according to the flowchart shown in Fig. 1. Radiologic images were analyzed in the current study according to the diagnostic criteria determined by the American Thoracic Society/European Thoracic Society. The committee gave not only evaluations of each individual case, but also advice on further investigations and safety measures. The committee was convened 10 times from November 2010 to December 2012.

If a reported term was contained within the Standard MedDRA Query (SMQ) “ILD” (narrow and broad), the event was required to be reviewed by the committee. If potential ILD events including pulmonary toxicities such as pneumonia, dyspnea, acute respiratory failure, and pulmonary edema that were not included in the SMQ “ILD” were indicated, these patients were assessed by the committee after a medical review.

If ILD or potential ILD was reported, detailed clinical information, including laboratory data, and treatment and medical histories were additionally collected and the reporting physicians were requested to submit the radiographic and computed tomography (CT) images.

### Statistical analysis

Exploratory multivariate analysis was performed to investigate risk factors for ILD. Two ILD events considered by the committee not to be related to panitumumab were also included in the analyses to avoid a bias. Any patient who developed ILD more than 10 months after the first administration was excluded. The potential variables were first selected based on the stratified frequency of ILD. The medical interests of ILD and dependency variables were taken into account, and the major variables were selected and multivariate analysis in a stepwise manner was performed using Cox’s proportional hazards model (Table 1). The following variables were contained as explanatory variables: sex, age, Eastern Cooperative Oncology Group Performance Status (ECOG PS), previous or concurrent ILD, previous drug treatment for colorectal cancer, a concomitant chemotherapy with FOLFOX, and smoking status. The statistical analysis was performed with SAS version 9.1 software.

**Table 1:** Method of multivariate analysis

Statistical method	Multivariate analysis using Cox’s proportional hazards model, stepwise process^a^, level of significance 5 %
Analysis population	2311 patients who had the full set of explanatory variables among 3085 patients who participated in the safety analysis
Response variable	
ILD (diagnosis by the ILD review subcommittee, adverse events)	Yes/no
Explanatory variable	
Sex	Male/female
Age	<65 years/≥65 years
ECOG PS	PS: 0, 1/PS: 2–4
Previous or concurrent ILD	No/yes
Previous drug treatment for colorectal cancer	No/yes
Concomitant chemotherapy FOLFOX	No/yes
Smoking status	No/yes (smoking and smoked in the past)

*ILD* interstitial lung disease, *ECOG PS* Eastern Cooperative Oncology Group Performance Status

^a^Gender, age, ECOG PS and smoking status were set as variables when a model was selected

## Results

### Patient characteristics

Forty-eight possible ILD case reports were evaluated and 39 events were determined to be panitumumab-induced ILD (Fig. 1). Patient demographics are shown in Table 2. Five of the 9 excluded patients were reported by primary physicians to have either a history of or concurrent ILD. In addition, 4 patients were excluded as they were considered by the committee, based on the evaluation of images, to have either a history or a complication of ILD, although the primary physicians reported that they had neither a history nor a complication of ILD.

**Table 2:** Patient demographics

Baseline characteristic	Safety analysis set (*N* = 3085)		ILD patients^a^ (*N* = 39)	
	*n*	%	*n*	%
Gender				
Male	1965	63.7	32	1.6
Female	1120	36.3	7	0.6
Age				
<65 years	1524	49.4	13	0.9
65–74 years	1058	34.3	16	1.5
≥75 years	503	16.3	10	2.0
Median (range)	65.0 (18–90)	–	69.0 (40–90)	
*KRAS* status				
Wild	3003	97.3	37	1.2
Mutant	3	0.1	1	33.3
Not determinable	79	2.6	1	1.3
ECOG PS				
0	1877	60.8	23	1.2
1	942	30.5	10	1.1
2	241	7.8	5	2.1
3	22	0.7	1	4.5
4	3	0.1	0	0.0
Treatment lines				
First line	310	10.1	11	3.5
Second line	543	17.6	8	1.5
Third line or later	2232	72.4	20	0.9
Past treatment regimens				
No	173	5.6	8	4.6
Yes (duplicate counting)	2911	94.4	31	1.1
FOLFOX	2439	79.1	25	1.0
FOLFIRI	1907	61.8	20	1.0
Bevacizumab	2113	68.5	23	1.1
Cetuximab	917	29.7	9	1.0
Others	2067	67.0	23	1.1
Unknown	1	0.0	0	0
Treatment regimen				
Monotherapy	1254	40.7	16	1.3
Chemotherapy (duplicate counting)	1831	59.4	23	1.3
FOLFOX	573	18.6	13	2.3
FOLFIRI	1045	33.9	13	1.2
CPT-11	277	9.0	0	0.0
Others	191	6.2	1	0.5
Smoking history				
No	1365	44.3	14	1.0
Yes	947	30.7	19	2.0
Current smoking	234	7.6	5	2.1
Smoked in the past	713	23.1	14	2.0
Unknown	773	25.1	6	0.8
Previous or concurrent ILD				
No	3051	98.9	34	1.1
Yes (reported by physicians)	34	1.1	5	14.7

*ECOG PS* Eastern Cooperative Oncology Group Performance Status, *ILD* interstitial lung disease

^a^Detected by the ILD review subcommittee

The total ILD frequency was 1.3 % (39/3085) [monotherapy group: 1.3 % (16/1254); combination therapy group: 1.3 % (23/1831); combination therapy with FOLFOX group: 2.3 % (13/573); combination therapy with FOLFIRI group: 1.2 % (13/1045); others: 0.5 % (1/191)]. The committee concluded that the mortality due to ILD was 51.3 % (20/39). Although the reporting physicians regarded that 3 patients among those who had ILD onset died due to their primary disease, it was considered that a causal relationship between the ILD and the death could not be ruled out by the committee. Therefore, they were included as fatal ILD.

### Imaging pattern

A diffuse alveolar damage (DAD) pattern was identified in 18 patients, including 5 patients who developed ILD with a non-DAD pattern at an earlier time point of panitumumab administration and progressed to ILD with DAD. Hypersensitivity (HP) and organizing pneumonia (OP) were also found in 9 and 8 patients, respectively. Two ILD patients whose imaging data were not provided were assessed based on the clinical course and laboratory data. The imaging patterns were categorized as “unknown.”

The mortality rate of patients with DAD was 83.3 % (15/18), while that of patients with non-DAD patterns, i.e., HP and OP, was 11.8 % (2/17) (Table 3).

**Table 3:** Number of ILD patients and length of time to the onset of ILD for each image pattern

Duration (months)	Total	≤1	≤2	≤3	≤4	≤5	≤6	≤7	≤8	≤9	≤10	>10
Number of patients	3085	2874	2570	2157	1774	1454	1210	1010	867	720	597	451
Number of ILD patients	39 (20)	8 (6)	4 (2)	11 (4)	1 (0)	4 (2)	5 (2)	0 (0)	3 (1)	2 (2)	0 (0)	1 (1)
Image pattern												
DAD	18 (15)	4 (4)	1 (1)	6 (4)	1 (0)	2 (2)	2 (2)	0 (0)	1 (1)	0 (0)	0 (0)	1 (1)
HP	9 (1)	1 (0)	1 (1)	3 (0)	0 (0)	1 (0)	1 (0)	0 (0)	2 (0)	0 (0)	0 (0)	0 (0)
OP	8 (1)	1 (0)	2 (0)	2 (0)	0 (0)	1 (0)	1 (0)	0 (0)	0 (0)	1 (1)	0 (0)	0 (0)
Unknown	4 (3)	2 (2)	0 (0)	0 (0)	0 (0)	0 (0)	1 (0)	0 (0)	0 (0)	1 (1)	0 (0)	0 (0)

Numbers of fatal cases are given in *parentheses*

*ILD* interstitial lung disease, *DAD* diffuse alveolar damage, *HP* hypersensitivity pneumonia, *OP* organizing pneumonia

### Timing of ILD onset

**Fig. 2:**
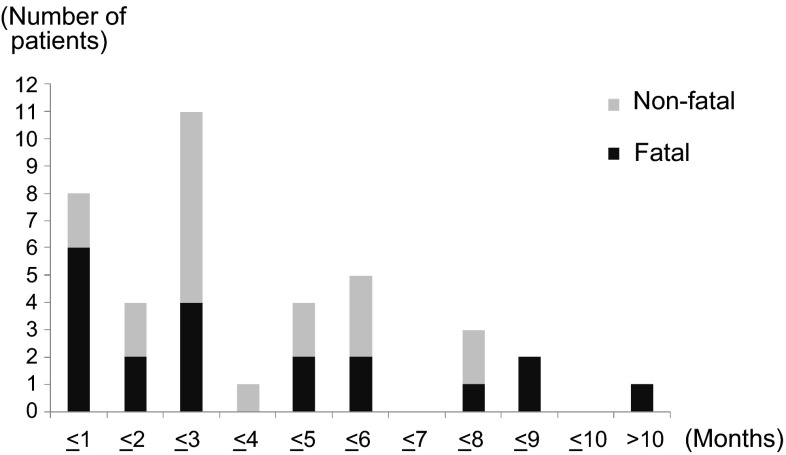
Time to onset of ILD. The numbers of ILD cases assessed by the ILD review subcommittee were classified by the length of time to the onset of ILD. Each outcome consists of non-fatal (*grey*) and fatal (*black*) cases. Note that there were no specific trends of ILD occurrence observed during the study. *ILD* interstitial lung disease

Figure 2 and Table 3 show the number of ILD patients and the length of time to the onset of ILD. No significant correlations were found between the timing of ILD onset and the imaging patterns (time to onset from start of administration: 2–313 days; 1–19 courses). In 11 patients, ILD occurred 6 months or longer after the first administration of panitumumab.

### Risk factors

Of the 3085 patients, 774 (25.1 %), including several who developed ILD, were excluded from the multivariate analysis because of unknown background information (information about a smoking history in 773 patients and a prior treatment history in 1 patient was not available). Therefore, only 2311 patients were used for the multivariate analysis. Of these patients, 34 were assessed to have ILD.

**Fig. 3:**
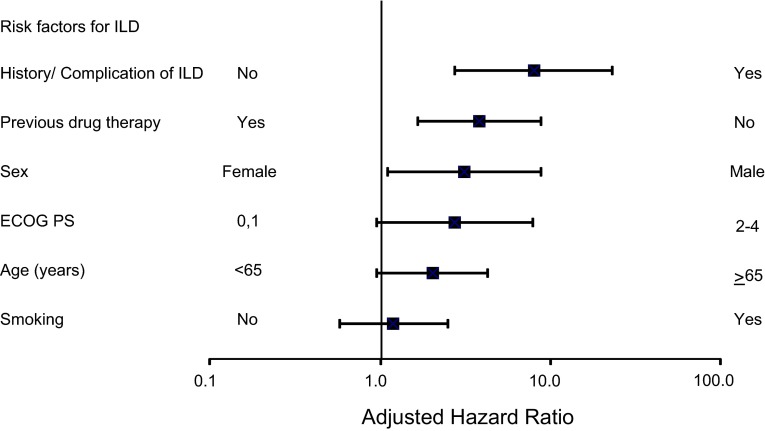
Forest plot of risk factors. By employing multivariate analysis using Cox’s proportional hazard model, higher risk factors of ILD occurrence were identified, and the adjusted HR is shown. *ILD* interstitial lung disease, *ECOG PS* Eastern Cooperative Oncology Group Performance Status, *HR* hazard ratio

Among the 34 patients, the risk of ILD occurrence was significantly higher in those with a history/complication of ILD, no history of previous drug treatment, and male sex (Table 4; Fig. 3). The adjusted hazard ratio (HR) was greater than 2.0 in the groups of 65 years or older and ECOG PS 2–4.

**Table 4:** Results of multivariate analysis

Background factor		No. of patients	Follow-up (person-months)	Patients with ILD	Incidence (%)	Incidence rate (/100 person-months)	Criterion variable	Explanatory variable	HR (unadjusted)	HR (adjusted)	95 % CI		*p* value
											Lower	Upper	
History/complication of ILD													
No		2283	15422	30	1.31	0.19	No	Yes	11.01	7.99	2.75	23.28	<0.001
Yes		28	188	4	14.29	2.13							
Sex													
Male		1399	9446	29	2.07	0.31	Female	Male	3.78	3.15	1.11	8.90	0.031
Female		912	6164	5	0.55	0.08							
ECOG PS													
PS: 0, 1		2104	14713	30	1.43	0.20	PS: 0, 1	PS: 2–4	2.27	2.74	0.95	7.91	0.062
PS: 2–4		207	897	4	1.93	0.45							
Age													
<65 years		1143	7740	10	0.87	0.13	<65	≥65	2.36	2.02	0.96	4.28	0.065
≥65 years		1168	7870	24	2.05	0.30							
Smoking history													
No		1364	9189	15	1.10	0.16	No	Yes	1.81	1.20	0.58	2.52	0.625
Yes		947	6421	19	2.01	0.30							
History of previous drug treatment of colorectal cancer													
No		131	948	7	5.34	0.74	Yes	No	3.99	3.83	1.66	8.84	0.002
Yes		2180	14662	27	1.24	0.18							

*ILD* interstitial lung disease, *HR* hazard ratio, *CI* confidence interval, *ECOG PS* Eastern Cooperative Oncology Group Performance Status

## Discussion

In order to manage and prevent fatal outcomes due to ILD, the current study focused on the clinical features and risk factors of panitumumab-induced ILD. Several postmarketing surveillance studies have suggested that the frequency of drug-induced ILD was higher in Japan than in other countries [13, 15, 16, 20, 21]. This difference might be due to many reasons: e.g., genetic susceptibility based on the different genetic background, underlying comorbidity, previous environmental exposures, clinical practices, frequency of diagnosis device, detection bias, and preference of reporting terms [15]. In the clinical trials of panitumumab monotherapy in metastatic colorectal cancer involving 1052 patients, there were no reports of adverse drug reactions in patients considered to have panitumumab-induced ILD, while in the clinical trials of the combination therapy with FOLFIRI [8] and with FOLFOX4 [7], adverse drug reactions considered to be panitumumab-induced ILD were reported at the rate of 0.7 % (2/302 patients; fatal, 0/2) and 0.6 % (2/322 patients; fatal due to ILD, 2/2), respectively. The incidence rate of ILD in this postmarketing all-case surveillance study in Japan was 1.3 % (39/3085 patients; fatal due to ILD, 20/39), which appeared slightly higher than that observed in premarketing clinical trials. Of the 20 patients who died due to ILD, 15 patients exhibited a DAD pattern and most of the patients were treated with steroid administration, but in 1 patient the treatment was considered to have been initiated late. Among the 20 deaths due to ILD, 3 out of 5 patients (including 3 non-evaluable patients) with a non-DAD pattern were treated with steroid administration, while the information regarding steroid treatment in the remaining 2 patients was missing. The ILD incidence and mortality rates were similar to those of the other anti-EGFR antibody [17, 20]. Data on the patients who died and those who did not were reviewed, but no particular differences in background factors or time to ILD onset, excluding a higher mortality rate in patients with a DAD pattern, were found between the 2 groups.

It is beneficial for clinicians to understand the clinical features of drug-induced ILD in daily medical practice. For instance, capillary leak syndrome by bortezomib administration [22] and high frequency of grade 1–2 ILD by mammalian target of rapamycin (mTOR) inhibitor [23] are examples of specific ILD features related to molecularly targeted drugs [16]. Panitumumab as well as cetuximab and EGFR-TKIs such as gefitinib and erlotinib can induce ILD with a DAD pattern, which can sometimes lead to a fatal outcome [24–26]. However, in this study, no findings specific to panitumumab were identified based on CT images or clinical practice.

It has been reported that an early onset of ILD occurrence was observed during EGFR-TKI administration [13]. Since no specific time-onset of ILD occurrence was found when panitumumab, as well as cetuximab, was administered, close and regular pulmonary monitoring is important for panitumumab administration.

From the results of the multivariate analysis, a history/complication of ILD, no history of previous drug treatment, and male sex were considered to be significant risk factors for panitumumab-induced ILDs. ECOG PS 2–4 and age of 65 years or older were also indicated as potential risk factors. These risk factors for ILD associated with panitumumab use are almost the same as those reported for the EGFR-TKIs [13, 14, 18, 19] and the other anti-EGFR monoclonal antibody [20]. No history of previous drug treatment could be an apparent risk factor because of patient selection bias. The reasons are as follows: 666 patients previously treated with the other anti-EGFR monoclonal antibody, cetuximab, were included in the 2180 patients with previous treatment. These patients were considered to be less likely to experience ILD even when treated with panitumumab (the incidence of ILD was 0.66 % [6/914] in patients who received panitumumab among patients who had received the other anti-EGFR monoclonal antibody and had not experienced ILD). In addition to the patient selection bias, it was unlikely that the patients undergoing first-line treatment had a higher risk of ILD than those undergoing second-line or later treatment, because, generally speaking, a patient undergoing second-line or later treatment had disease progression and their general condition was worse. Similarly, the incidence of ILD was significantly higher in elderly patients and those who had prior ILD, according to the results of postmarketing all-case surveillance of cetuximab in Japan [20]. In this study, cetuximab was used as second-line or later therapy after some chemotherapy [17]. The risk factors for ILD occurrence during therapy with an anti-EGFR monoclonal antibody need further investigation.

The following 3 factors are important for detecting ILD at an early stage: (1) examining whether patients have signs or symptoms such as dry cough, dyspnoea, and pyrexia which suggest the patients have ILD, and consulting with pulmonologists at an early stage if the signs or symptoms are found; (2) informing patients or their family of signs or symptoms of ILD in advance and counseling the patients to see a physician and report the signs or symptoms immediately after onset; and (3) examining images of patients at the following times: (a) when physicians evaluate whether patients have lung metastasis before panitumumab is administered; (b) when the efficacy of panitumumab is evaluated; and (c) when signs or findings that show patients are suspected of having ILD are obtained.

The following are considered to be limitations in the current analysis: (A) the all-case surveillance was not designed to identify ILD risk factors, and therefore only selected risk factors (listed in Table 4) were taken into consideration; (B) adverse events of both the monotherapy and combination chemotherapy were analyzed together, and thus impacts of the combination chemotherapy could not be excluded; and (C) since 773 patients without information on smoking, a risk factor of ILD, were excluded from the analysis, the impact of smoking could not be fully considered. A benefit–risk balance must be thoroughly considered before giving the drug to patients with interstitial pneumonia or lung fibrosis or those who have histories of them, and careful decisions must be made when deciding to initiate panitumumab.

In conclusion, the ILD incidence mortality rates and risk factors of panitumumab were similar to those of the other anti-EGFR monoclonal antibody [17, 20]. Panitumumab-specific ILD findings were not observed in CT images or clinical practice. Panitumumab likely induces DAD just as the other anti-EGFR monoclonal antibody and EGFR-TKIs do, which may lead to death. A history/complication of ILD, male sex, poor general condition, and age of 65 years or older were indicated to be ILD risk factors in the multivariate analysis, and these are similarly observed in the reports with the EGFR-TKIs and anti-EGFR monoclonal antibody [13, 14, 20]. In addition, no history of previous drug treatment was considered to be an apparent risk factor. ILD can occur at any time after initiating panitumumab, and therefore close and regular monitoring is needed. Although several papers show that the frequency of EGFR-TKI-induced ILD was higher in Japan than in Western countries [12, 18, 19], the reason was unclear. Some studies suggest that genetic differences make a contribution [27, 28]. Anti-EGFR monoclonal antibodies might have similar mechanisms to EGFR-TKIs, and therefore ILD incidence is higher in Japan. Future studies are clearly warranted to investigate the benefit–risk balance in patients with the risk factors identified in this study.

## References

[1] Yang XD, Jia XC, Corvalan JR (2001). Development of ABX-EGF, a fully human anti-EGF receptor monoclonal antibody, for cancer therapy. Crit Rev Oncol Hematol.

[2] Van Cutsem E, Peeters M, Siena S (2007). Open-label phase III trial of panitumumab plus best supportive care compared with best supportive care alone in patients with chemotherapy-refractory metastatic colorectal cancer. J Clin Oncol.

[3] Hecht JR, Patnaik A, Berlin J (2007). Panitumumab monotherapy in patients with previously treated metastatic colorectal cancer. Cancer.

[4] Amado RG, Wolf M, Peeters M (2008). Wild-type *KRAS* is required for panitumumab efficacy in patients with metastatic colorectal cancer. J Clin Oncol.

[5] Weiner LM, Belldegrun AS, Crawford J (2008). Dose and schedule study of panitumumab monotherapy in patients with advanced solid malignancies. Clin Cancer Res.

[6] Siena S, Peeters M, Van Cutsem E (2007). Association of progression-free survival with patient-reported outcomes and survival: results from a randomised phase 3 trial of panitumumab. Br J Cancer.

[7] Douillard JY, Siena S, Cassidy J (2010). Randomized, phase III trial of panitumumab with infusional fluorouracil, leucovorin, and oxaliplatin (FOLFOX4) versus FOLFOX4 alone as first-line treatment in patients with previously untreated metastatic colorectal cancer: the PRIME study. J Clin Oncol.

[8] Peeters M, Price TJ, Cervantes A (2010). Randomized phase III study of panitumumab with fluorouracil, leucovorin, and irinotecan (FOLFIRI) compared with FOLFIRI alone as second-line treatment in patients with metastatic colorectal cancer. J Clin Oncol.

[9] Muro K, Yoshino T, Doi T (2009). A phase 2 clinical trial of panitumumab monotherapy in Japanese patients with metastatic colorectal cancer. Jpn J Clin Oncol.

[10] Boku N, Sugihara K, Kitagawa Y (2014). Panitumumab in Japanese patients with unresectable colorectal cancer: a post-marketing surveillance study of 3085 patients. Jpn J Clin Oncol.

[11] Horiuchi-Yamamoto Y, Gemma A, Taniguchi H (2013). Drug-induced lung injury associated with sorafenib: analysis of all-patient post-marketing surveillance in Japan. Int J Clin Oncol.

[12] Barber NA, Ganti AK (2011). Pulmonary toxicities from targeted therapies: a review. Target Oncol.

[13] Kudoh S, Kato H, Nishiwaki Y (2008). Interstitial lung disease in Japanese patients with lung cancer: a cohort and nested case-control study. Am J Respir Crit Care Med.

[14] Nakagawa K, Kudoh S, Ohe Y (2012). Postmarketing surveillance study of erlotinib in Japanese patients with non-small-cell lung cancer (NSCLC): an interim analysis of 3488 patients (POLARSTAR). J Thorac Oncol.

[15] Koo LC, Clark JA, Quesenberry CP (2005). National differences in reporting pneumonia and pneumonia interstitial: an analysis of the WHO International Drug Monitoring Database on 15 drugs in nine countries for seven pulmonary conditions. Pharmacoepidemiol Drug Saf.

[16] Saito Y, Gemma A (2012). Current status of DILD in molecular targeted therapies. Int J Clin Oncol.

[17] Ishiguro M, Watanabe T, Yamaguchi K (2012). A Japanese post-marketing surveillance of cetuximab [Erbitux(R)] in patients with metastatic colorectal cancer. Jpn J Clin Oncol.

[18] Ando M, Okamoto I, Yamamoto N (2006). Predictive factors for interstitial lung disease, antitumor response, and survival in non-small-cell lung cancer patients treated with gefitinib. J Clin Oncol.

[19] Gemma A (2009). Drug-induced interstitial lung diseases associated with molecular-targeted anticancer agents. J Nippon Med Sch.

[20] Sato T, Gemma A, Kudoh S (2014). Incidence and clinical features of drug-induced lung injury in patients with advanced colorectal cancer receiving cetuximab: results of a prospective multicenter registry. Jpn J Clin Oncol.

[21] Raghu G, Nyberg F, Morgan G (2004). The epidemiology of interstitial lung disease and its association with lung cancer. Br J Cancer.

[22] Mukai H, Ohyashiki K, Katoh T (2011). Lung injury associated with bortezomib therapy in Japan. Rinsho Ketsueki.

[23] Noguchi S, Masuda N, Iwata H (2014). Efficacy of everolimus with exemestane versus exemestane alone in Asian patients with HER2-negative, hormone-receptor-positive breast cancer in BOLERO-2. Breast Cancer.

[24] Inoue A, Saijo Y, Maemoto M (2003). Severe acute interstitial pneumonia and gefitinib. Lancet.

[25] Makris D, Scherpereel A, Copin MC (2007). Fatal interstitial lung disease associated with oral erlotinib therapy for lung cancer. BMC Cancer.

[26] Camus P, Kudoh S, Ebina M (2004). Interstitial lung disease associated with drug therapy. Br J Cancer.

[27] Seibold MA, Wise AL, Speer MC (2011). A common MUC5B promoter polymorphism and pulmonary fibrosis. N Engl J Med.

[28] Cronkhite JT, Xing C, Raghu G (2008). Telomere shortening in familial and sporadic pulmonary fibrosis. Am J Respir Crit Care Med.

